# Unprofessional Conduct of an Army Surgeon

**Published:** 1898-09

**Authors:** 


### UNPROFESSIONAL CONDUCT OF AN ARMY SURGEON.

Dr. G. A. Baxter has preferred charges of conduct unbecoming an officer and a gentleman against Major Surgeon Samuel D. Hubbard, of the Ninth New York Regiment. The specifications are that Dr. Hubbard "cursed and swore" at Dr. Baxter and other Chattanooga physicians who had been called to attend Sergeant Frank, of the Ninth New York, who was recently injured under the wheels of a moving railroad train; that Hubbard had removed Frank, who was in a state of profound shock from his injuries, against the protest of Baxter and other physicians, and that as a consequence Frank died on his way to the division hospital, losing the only chance he had of recovery by being improperly handled." The charges were filed with General Breckinridge to-day and an investigation and trial will be ordered—.*Charlotte Medical Journal.*

